# Identification of chromosomal abnormalities in miscarriages by CNV-Seq

**DOI:** 10.1186/s13039-024-00671-7

**Published:** 2024-02-18

**Authors:** Yuqi Shao, Saisai Yang, Lin Cheng, Jie Duan, Jin Li, Jiawei Kang, Fang Wang, Juan Liu, Fang Zheng, Jianhong Ma, Yuanzhen Zhang

**Affiliations:** 1 Department of Obstetrics, Zhongnan Hospital of Wuhan University, Wuhan, 430071 China; 2 Hubei Clinical Research Center for Prenatal Diagnosis and Birth Health, Wuhan, 430071 China; 3 Wuhan Clinical Research Center for Reproductive Science and Birth Health, Wuhan, 430071 China; 4 Center for Gene Diagnosis, Department of Clinical Laboratory Medicine, Zhongnan Hospital of Wuhan University, Wuhan, 430071 China

**Keywords:** Chromosomal abnormalities, Copy number variants, Miscarriage, CNV-Seq

## Abstract

**Objective:**

The primary object of this study is to analyze chromosomal abnormalities in miscarriages detected by copy number variants sequencing (CNV-Seq), establish potential pathways or genes related to miscarriages, and provide guidance for birth health in the following pregnancies.

**Methods:**

This study enrolled 580 miscarriage cases with paired clinical information and chromosomal detection results analyzed by CNV-Seq. Further bioinformatic analyses were performed on validated pathogenic CNVs (pCNVs).

**Results:**

Of 580 miscarriage cases, three were excluded as maternal cell contamination, 357 cases showed abnormal chromosomal results, and the remaining 220 were normal, with a positive detection rate of 61.87% (357/577). In the 357 miscarriage cases, 470 variants were discovered, of which 65.32% (307/470) were pathogenic. Among all variants detected, 251 were numerical chromosomal abnormalities, and 219 were structural abnormalities. With advanced maternal age, the proportion of numerical abnormalities increased, but the proportion of structural abnormalities decreased. Kyoto Encyclopedia of Genes and Genomes pathway and gene ontology analysis revealed that eleven pathways and 636 biological processes were enriched in pCNVs region genes. Protein–protein interaction analysis of 226 dosage-sensitive genes showed that *TP53*, *CTNNB1*, *UBE3A*, *EP300*, *SOX2*, *ATM*, and *MECP2* might be significant in the development of miscarriages.

**Conclusion:**

Our study provides evidence that chromosomal abnormalities contribute to miscarriages, and emphasizes the significance of microdeletions or duplications in causing miscarriages apart from numerical abnormalities. Essential genes found in pCNVs regions may account for miscarriages which need further validation.

**Supplementary Information:**

The online version contains supplementary material available at 10.1186/s13039-024-00671-7.

## Introduction

Miscarriage is spontaneous pregnancy loss that happens before 28 gestational weeks and can be divided into early miscarriage and late miscarriage according to a boundary of 12 gestational weeks. As reported, the incidence of miscarriage is about 15–20%, with an increasing trend year by year [1]. About 1% of couples have experienced repeated pregnancy losses, mainly in early pregnancy [2]. The causes of miscarriage are complex, and the recognized reasons mostly comprise genetic factors, maternal factors, and environmental factors [3, 4].

Studies have revealed that chromosome aneuploidies and polyploidies are frequently detected in miscarriages, especially in early miscarriages, with an approximate prevalence of 50% [2, 3]. Further studies have also discovered copy number variants (CNVs) in miscarriages, and a systematic analysis has revealed the relationship between CNVs and miscarriages [5, 6]. CNVs refer to deletions or duplications of genome segments, some CNVs may contain numerous genes or overlay with regions of identified chromosomal disorders, but how CNVs result in miscarriages is still unclear.

Commonly, genetic analysis of abortion tissues mainly includes chromosome karyotype and chromosome microarray analysis (CMA). In recent years, with the advance of the next generation sequencing, CNV-Seq has been frequently applied in detecting chromosomal abnormalities clinically. CNV-Seq can find chromosome aneuploidies, polyploidies, microduplications, and microdeletions. Compared to karyotype and CMA, CNV-Seq is more high-resolution and more comprehensive in the detection of variants [7–9]. In addition, the cost of CNV-Seq is lower than CMA.

In this study, we systematically evaluated the chromosomal abnormalities of early and middle trimester abortion by CNV-Seq. We clarified the relationship between chromosomal abnormalities and miscarriages. Moreover, we analyzed the critical regions of CNVs detected to identify potential candidate genes related to miscarriages and further performed functional gene analysis using gene enrichment and protein interaction analysis. This study may help to screen the genetic causes of aborted fetuses and provide reproductive guidance for women of childbearing age.

## Materials and methods

### Subjects

580 spontaneous abortions that happened between 5 and 28 gestational weeks from January 2018 to December 2021 were enrolled in this study. The Medical Ethics Committee of Zhongnan Hospital of Wuhan University approved this study (Approval Number: 2023015K), and the written informed consent of all patients was obtained.

### Detection of chromosomal abnormalities

10 mg products of concept (POCs) were collected for chromosomal abnormalities detection, and maternal peripheral blood was collected for maternal contamination exclusion. DNA was extracted by a Qiagen DNA Blood Midi/Mini kit (Qiagen GmbH, Hilden, Germany), the purity and yield of DNA products were determined using NanoDrop spectrophotometer and agarose gel electrophoresis. STR analysis was conducted by a commercial kit (Microread, Beijing, China) before CNV-Seq, selected STR analysis markers were D19S433, D5S818, D6S1043, AMEL, D3S1358, D7S820, D16S539, CSF1PO, Penta, D2S441, D8S1179, FGA, D2S1338 and D13S317. In the end, 577 samples went on CNV-Seq, and the other three were excluded for maternal contamination. The qualified DNA genome was randomly broken into an average size of 200 bp, and the library was constructed by terminal repair, connector connection, and other methods using the high-throughput sequencing library kit of Beijing Berry. The NextSeq 500 platform (Illumina, San Diego, USA) was used to perform massively parallel sequencing with a reading length of 36 bp and a reading depth of 0.5×, which was a single-end sequencing. GRCh37/hg19 was the reference sequence for further analysis, and only CNVs larger than 100 kb were reported. Database of Genomic Variants (DGV)(16), DECIPHER (https://www.deciphergenomics.org/), OMIM (https://omim.org/®), and PubMed (https://pubmed.ncbi.nlm.nih.gov/) were explored for CNVs mapping and identification. For further exploration of the effects of CNVs beyond aneuploidies and polyploidies in miscarriages, we classified the variants as either numerical or structural abnormalities. Aneuploidies and polyploidies were categorized as numerical abnormalities, whereas deletions or duplications were classified as structural abnormalities. According to the American College of Medical Genetics and Genomics (ACMG) technical standards, structural abnormalities were classified into five categories: pathogenic CNVs (pCNVs), likely pathogenic CNVs (lpCNVs), variants of uncertain significance (VOUS), likely benign CNVs (lbCNVs), and benign CNVs (bCNVs) [10].

### Function analysis of potential genes

The UCSC Genome Browser (http://www.genome.ucsc.edu/) and ClinGen (https://www.clinicalgenome.org/) were used to locate genes found in chosen CNVs. Kyoto Encyclopedia of Genes and Genomes (KEGG) pathway and Gene Ontology (GO) analyses were performed using the “clusterProfiler” package and “ggplot2” package in R software (4.2.2) [11]. STRING (https://cn.string-db.org/) and Cytoscape were used to examine protein–protein interactions (PPI) [12, 13].

### Statistical analysis

Comparisons between groups were conducted using the chi-square test, which was performed in SPSS Statistics software v26.0 (IBM Corp, Armonk, NY). *P* < 0.05 was considered statistically significant.

## Results

### Subject characteristics

**Fig. 1:**
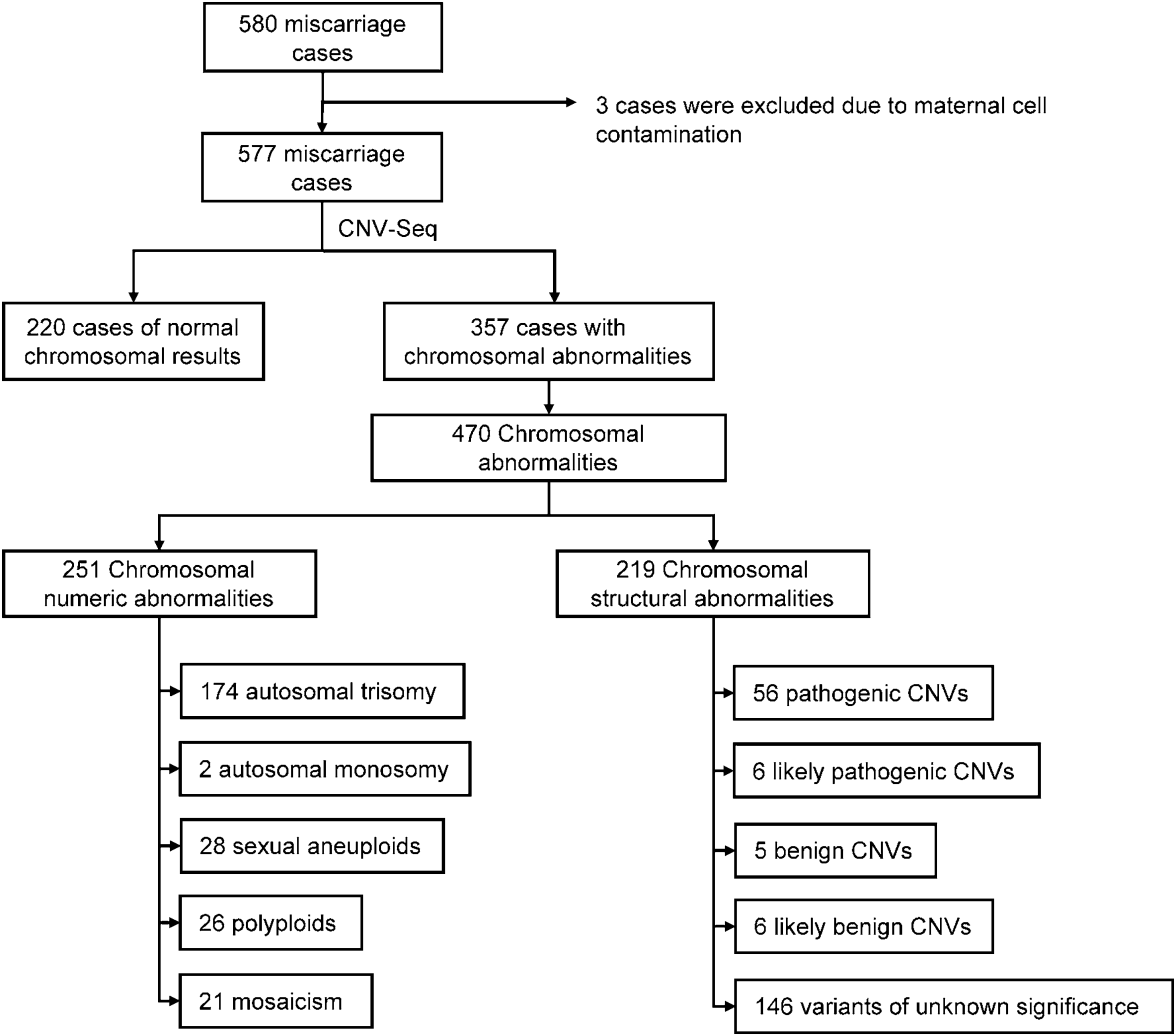
Study flowchart

A total of 580 abortion tissue samples were collected in this study, of which 3 samples were excluded due to maternal cell contamination, and CNV-Seq tested the remaining 577 samples (Fig. 1). The average gestational age at the time of CNV abnormal fetal abortion was 9.58 ± 2.93 weeks, ranging from 5 to 28 weeks. The average age of pregnant women was 31.17 ± 4.33 years old, ranging from 22 to 44 years old. 269 (46.62%) pregnant women were younger than 30 years old, 207 (35.88%) were 30–35 years old, 72 (12.48%) were 35–40 years old, and 29 (5.03%) were older than 40 years old.

### Chromosomal aberrations detected in miscarriages

**Fig. 2:**
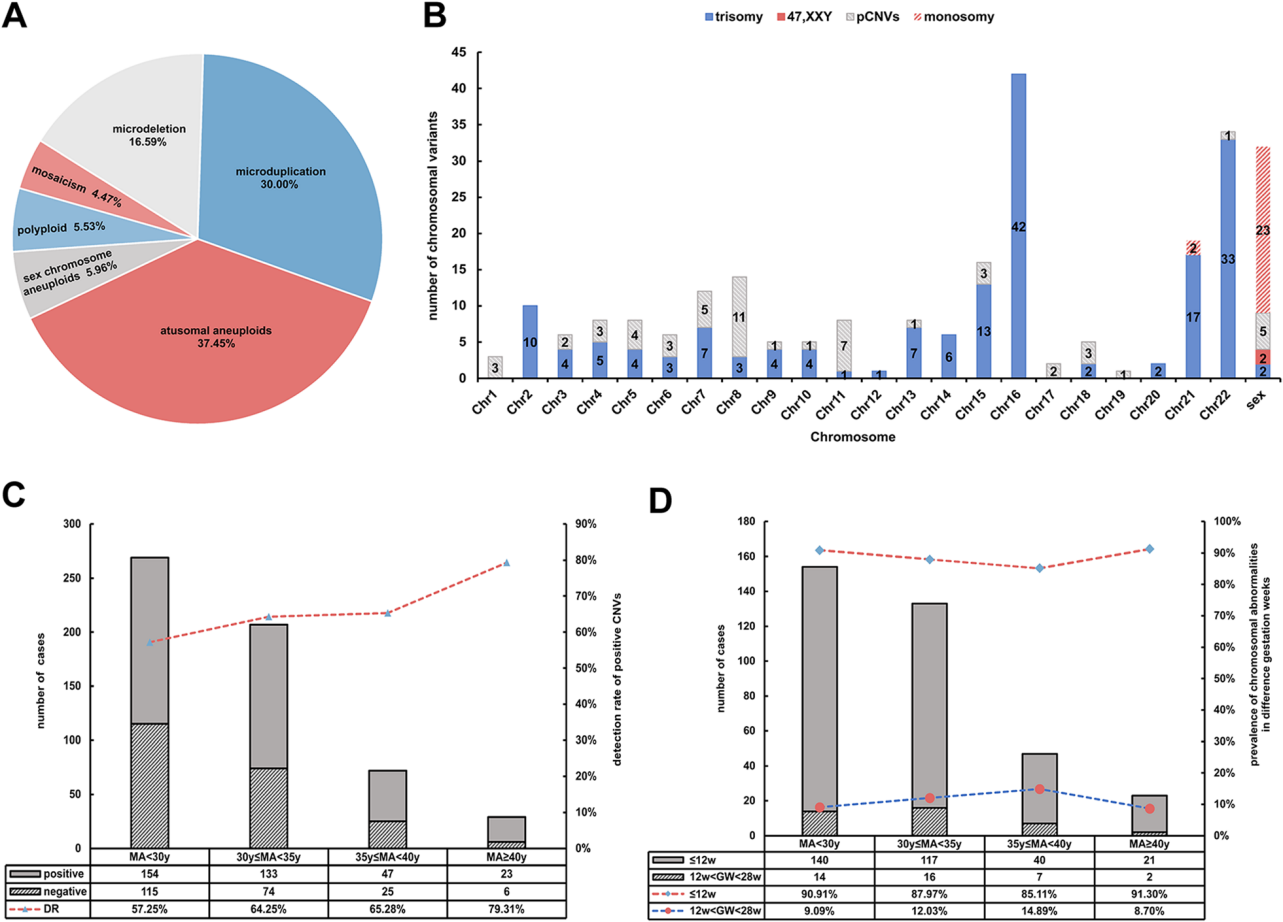
Distribution of chromosomal abnormalities. (**A**) General classification of variants with respective proportions. (**B**) The number of aneuploid and P CNVs in different chromosomes. (**C**) Distribution of cases with positive or negative chromosomal results in various maternal age groups. (**D**) The bar plot showed the distribution of early or late miscarriage in various maternal age groups and the line chart showed the trends of chromosomal abnormalities detected in different gestational weeks as the maternal age increased

Chromosomal abnormalities were found in 357 (61.87%) out of the 577 miscarriage cases, whereas the remaining 220 were normal (Table 1). 324 (90.76%) fetuses that had chromosomal abnormalities were aborted during the first trimester (gestational weeks, GW ≤ 12 weeks, and 33 (9.24%) were aborted during the middle trimester (12 < GW < 28). Numerical chromosomal abnormalities were detected in 69% (243/357) abortions (Fig. 2A), among them, 5 cases also exhibited additional complications with pCNVs (shown in Table 1- Cases with variants). In cases detected with structural abnormalities, pCNVs and VOUS were detected in most cases. In total, 470 variants were found, of which 54.40% (251/470) were numerical chromosomal abnormalities (Table 1). The frequent chromosomes that occurred in numerical abnormalities were chromosome 2 (Chr2), Chr13, Chr15, Chr16, Chr21, Chr22, and sex chromosomes (Fig. 2B). In addition to sex chromosomes, where monosomy happened most frequently, the other six chromosomes described above as well as Chr14 were frequent sites for trisomy. The 21 mosaicisms were listed in Additional file 2: Table S2.

**Table 1:** Baseline characteristics of cases with chromosomal abnormalities

Characters	Numbers	Proportion (%)	Sum (n)
^A^Gestation weeks (GW, 9.58 ± 2.93 w)			
GW ≤ 12 w	324	90.76	357
12w < GW < 28 w	33	9.24	
Maternal age (MA, 31.17 ± 4.33 y)			
MA ≤ 30 y (27.49 ± 1.60)	154	43.14	357
30 y < MA < 35 y (31.66 ± 1.34)	133	37.25	
35y ≤ MA < 40 y (36.41 ± 1.26)	47	13.17	
MA ≥ 40 y (41.72 ± 1.61)	23	43.14	
^B^Number of cases with variants			
Cases with numerical abnormalities	^C^243		357
Autosomal trisomy	166	68.31	
Autosomal monosomy	2	0.82	
Triploidy	24	9.88	
Monosomy X	25	10.29	
47, XXX/47, XXY	2	0.82	
Trisomy 22, 47, XXY	1	0.41	
Tisomy 13, 45, X	1	0.41	
Mosaicism	21	8.64	
Cases with structural abnormalities	^C^119		
pCNVs	31	26.05	
lpCNVs	4		
bCNVs	5	4.20	
lbCNVs	0	–	
VOUS	79	66.39	
Variants			
Microdeletion	78	16.60	470
Microduplication	141	30.00	
Autosomal trisomy	174	37.02	
Autosomal monosomy	2	0.43	
Sex chromosome aneuploids	28	5.96	
Polyploid	26	5.53	
Mosaicism	21	4.47	
Pathogenic classification			
Pathogenic	307	65.32	470
Likely pathogenic	6	1.28	
Benign	5	1.06	
Likely benign	6	1.28	
VOUS	146	31.06	

^A^Mean ± standard deviation

^B^In this part, cases were grouped into cases with numerical abnormalities (aneuploidies, polyploidies and mosaicism) or cases with structural abnormalities (deletions or duplications). Structural abnormalities were classified as pCNVs, lpCNVs, bCNVs, lbCNVs and VOUS according to ACMG guidelines

^C^Of the 357 cases detected with variants, 5 cases were detected with both numerical abnormalities and structural abnormalities, the 5 cases were represented twice in this table

Furthermore, 219 structural chromosomal abnormalities were found with a proportion of 46.60% (219/470), pCNVs made up 11.91% (56/470) of variations, lpCNVs made up 1.28% (6/470), bCNVs made up 1.06% (5/470), lbCNVs made up 1.28% (6/470), and VOUS made up 31.06% (146/470). CNV size ranged from 0.10 to 136.02 Mb. In pCNVs and VOUS, variants less than 1.0 Mb accounted respectively for 8.93% (5/56) and 89.73% (131/146), and variants of 1.0–10.0 Mb accounted respectively for 26.79% (15/56) and 10.27% (15/146). The majority of pCNVs were larger than 10.0 Mb with a proportion of 64.28% (36/56). Of the 56 pCNVs detected in 31 POCs, 15 kinds of identified chromosomal disorders were overlayed (Additional file 1: Table S1, Additional file 3: Table S3).

### Comparison of CNVs among pregnancies of different maternal ages or gestational weeks

To analyze the association between various chromosomal abnormalities and maternal age (MA) in miscarriages, we divided pregnancies into 4 groups: MA < 30 y, 30 y ≤ MA < 35 y, 35 y ≤ MA < 40 y, and MA ≥ 40 y, the related kinds of variants were numerical chromosomal abnormalities and structural abnormalities (Table 2). For detailed analysis of structural abnormalities in different groups, variants were also grouped into pCNVs, lpCNVs, bCNVs, lbCNVs and VOUS, however, apart from pCNVs and VOUS the sizes of the remaining three groups were too small for effective statistical analyses. The fraction of abnormal chromosome numbers steadily rose with MA (Fig. 2C), with a significant statistical difference (χ^2^ = 25.379, *P* < 0.05). The ratio of structural chromosomal abnormalities gradually decreased with the increasing age of pregnant women, with a statistically significant difference (χ^2^ = 11.722, *P* < 0.05).

**Table 2:** Statistics of cases with chromosomal abnormalities in various maternal age groups and gestational age groups

Characters	Maternal age (MA)						Gestational weeks (GW)			
	^A^MA < 30 y	^B^30 y ≤ MA < 35 y	^C^35 y ≤ MA < 40 y	MA ≥ 40 y	χ2	*P* value	^D^GW ≤ 12w	12 w < GW ≤ 28 w	χ^2^	*P* value
Numerical chromosomal abnormalities	90 (1)	89 (3)	41 (1)	23	25.379	< 0.05	228 (5)	15	7.466	< 0.05
Structural chromosomal abnormalities	65 (1)	47 (3)	7 (1)	0	11.722	< 0.05	101 (5)	18	6.348	< 0.05
pCNVs	18 (1)	11 (3)	2 (1)	0	0.285	0.867	28	3	0.480	0.488
VOUS	43	32	4	0	0.330	0.848	68	11	0.059	0.808

^A^In the group of MA < 30 y, 1 case was detected with both numerical and structural abnormalities, the structural abnormalities in this case detected were classified as pCNVs, this case was represented three times in this table. The number in the bracket represents the case mentioned, the same in below

^B^In the group of 30 ≤ MA < 35 y, 3 cases were detected with both numerical and structural abnormalities, the structural abnormalities detected in these cases were classified as pCNVs, these cases were represented three times in this table

^C^In the group of 35 ≤ MA < 40 y, 1 case was detected with both numerical and structural abnormalities, the structural abnormalities in this case detected were classified as pCNVs, this case was represented three times in this table

^D^In the group of GW ≤ 12 w, 5 cases were detected with both numerical and structural abnormalities

To analyze the relationship between different chromosomal abnormalities and gestation weeks (GW), we divided pregnancies into to groups: early gestation week (GW ≤ 12 w) and middle gestation week (12 w < GW ≤ 28 w), variants were grouped as above (Table 2). Most of miscarriages happened during early stage of gestation might be the result of chromosomal abnormalities. As for specific kinds of variants, such as pCNVs or VOUS, there was no difference between GW.

### Potential candidate genes and signaling pathways analyzed in miscarriages

**Fig. 3:**
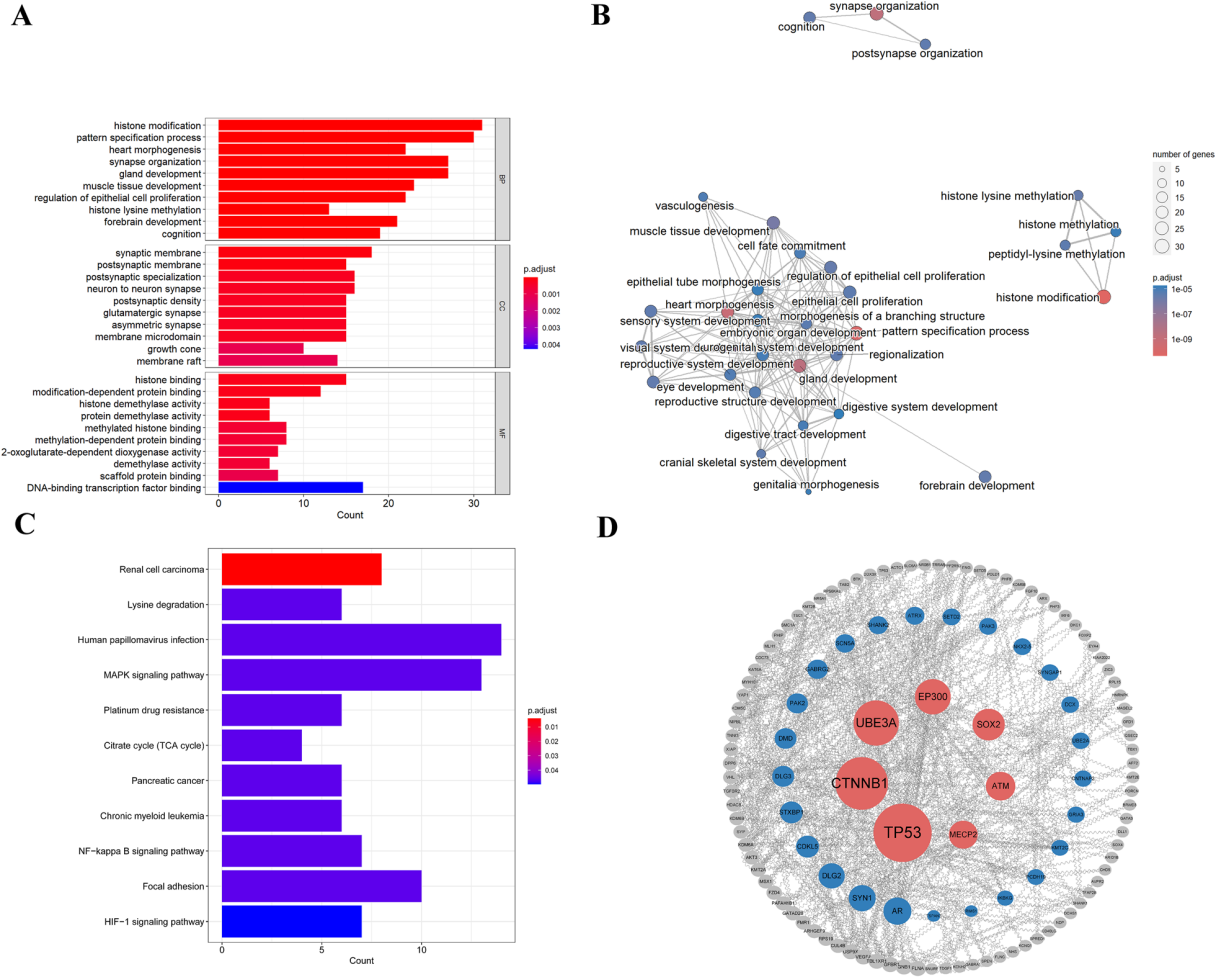
GO, KEGG analysis and PPI network of selected genes. (**A** and **B**) GO analysis of 226 genes (*P* < 0.05). (**C**) Eleven enriched pathways in KEGG analysis (*P* < 0.05). (**D**) *TP53*, *CTNNB1*, *UBE3A*, *EP300*, *SOX2* and *ATM* were selected to be significant genes in PPI network

KEGG pathway analysis revealed that the genes located in pCNVs significantly clustered in the following eleven pathways (Fig. 3C), renal cell carcinoma (*P* = 1.68E−05), lysine degradation (*P* = 5.86E−04), human papillomavirus (*P* = 1.15E−03), MAPK signaling pathway (*P* = 1.16E−03), platinum drug resistance (*P* = 1.28E−03), citrate cycle (TCA cycle) (*P* = 1.43E−03), pancreatic cancer (*P* = 1.58E−03), chronic myeloid leukemia (*P* = 1.58E−03), NF-κB signaling pathway (*P* = 1.66E−03), focal adhesion (*P* = 1.84E−03) and HIF-1 signaling pathway (*P* = 2.18E−03).

The GO results showed that the genes were enriched in 31 molecular functions (MF), 33 cellular components (CC), and 636 biological processes (BP), respectively. The most significant BP were histone modification (*P* = 4.59E−11), followed by pattern specification process (*P* = 1.35E−10), heart morphogenesis (*P* = 7.32E−10), synapse organization (*P* = 1.96E−09) and so on (Fig. 3A). BP network analysis showed that the most enriched biological processes were associated with organ development (e.g., heart morphogenesis, forebrain development, embryonic organ development) (Fig. 3B).

There are 226 dosage-sensitive genes in 56 pCNVs (Additional file 1: Table S1). As shown in Fig. 3D, we analyzed the association among genes selected according to their betweenness. With higher betweenness, larger nodes were shown. PPI network analysis revealed seven hub genes in miscarriages as *TP53*, *CTNNB1*, *UBE3A*, *EP300*, *SOX2*, *ATM*, and *MECP2*.

## Discussion

Miscarriage is a frequent adverse event in pregnancies for various reasons. The causes of miscarriages include embryonic genetic defects [14], maternal systemic diseases, genital abnormalities [15], endocrine abnormalities [16], immune abnormalities [17], fetal abnormalities, and some other factors [18]. In this study, we analyzed and discussed the contribution of chromosomal abnormalities in miscarriages. CNV-Seq was the chosen technology for detecting variants. The further analysis explored the influences of maternal age and gestational weeks in detecting chromosomal abnormalities in miscarriages. Meanwhile, KEGG pathway analysis, GO analysis, and PPI analysis were used for bioinformatic analysis of genes in pCNVs, aiming to figure out potential occurrence mechanisms or candidate genes for miscarriages.

577 POCs of miscarriages were analyzed by CNV-Seq. The positive rate of chromosomal abnormality was 61.87%, slightly higher than previous studies [19, 20], and the normal rate was 38.13%. A total of 470 variants were detected among 357 miscarriage cases, numerical chromosomal abnormalities accounted for 54.50%, and structural chromosomal abnormalities accounted for 46.60%. Of the 251 numerical chromosomal abnormalities, 174 autosomal trisomy were founded in 166 cases. Agreeing with previous studies, we found that trisomy mainly occurred in Chr2 (5.75%, 10/174), Chr15 (9.20%, 16/174), Chr21 (9.20%, 16/174), Chr22 (19.54%, 34/174) and Chr16 (24.71%, 43/174) with an increasing frequency [21, 22]. As reported, trisomy 16 (T16) is the most common in early abortion, with a proportion of 18–30%, resulting in about 6% of early abortions [23, 24]. We also found that no abnormal chromosome number was detected in Chr1, Chr17, and Chr19, which might result from the insufficient sample size. The incidence of miscarriages caused by T1, T17, and T19 was relatively low, although several studies found numerical abnormalities of Chr1 and Chr19 in abortion tissues [25, 26]. There were 21 mosaicisms detected in this study, and three were low-level mosaicism: 47,XNN[15%]/46,XN[85%]; 45,X[10%]/46,XN[90%]; 47,XN, + 4[10%]/46,XN[90%]. Ma et al. retrospectively compared the evaluating accuracy and efficiency among karyotype, CMA, and CNV-Seq in detecting mosaicism. The results show that CNV-Seq performs better in detecting low-level mosaicism with a detection limit of down to 5% [27].

It's well-recognized that advanced maternal age is associated with miscarriage and fetal aneuploidy [28].With advanced maternal age, ovarian functions and egg qualities decline, which results in chromosome non-separation or aberration of germ cells and fertilized eggs during meiosis [29]. Consistent with other studies, the proportion of numerical abnormalities rose as raised MA with statistical significance. As for structural abnormalities, a decreasing trend was found when maternal age increased, which also agreed with previous reports [30]. However, some studies argued that the frequency of structural abnormalities seems to be unrelated to the maternal age [31], thus, large amounts of data are urgently needed for further analysis. In addition to advanced maternal age, our results also suggest that abortions occurring in early gestation might contribute to chromosomal abnormalities, in agreement with previous studies [30].

The contribution of numerical chromosomal abnormalities was discussed above, the following was the discussion of structural abnormalities. Of the 219 structural variants, 56 were pCNVs (11.91%, 56/470), and 146 (31.06%, 146/470) were VOUS. The detection rate of pCNVs (5.37%, 31/577) was consisted with reported at 2.2–13%, whereas the detection rate of VOUS was higher compared with other studies [32]. We suspected that the inconsistent detection rate of VOUS might be a consequence of missing parental validation. Among 56 pCNVs, we found four confirmed regions related to miscarriages as 3q29 microduplication, 1p36 microdeletion, 5p deletion, and 8p23.1 deletion. In this cohort, 7q36.3 was frequent region (5/56, 8.83%) where pCNVs detected. As reported, deletion or duplication of 7q36.3 may result in developmental delay and congenital heart disease [33]. There are 16 protein-coding genes located in this region, among them, expression of the *SHH* gene was confirmed as impaired in the villus of recurrent miscarriages which means that dysfunction of *SHH* might link to miscarriages [34]. We surfed previous studies, finding that deletions or duplications in 7q36.3 were detected in several POCs [6, 35, 36]. Taken together, we hypothesized that 7q36.3 might be a candidate region related to miscarriages, for which more evidence is urged to indicate the association.

It’s still a mystery how CNVs lead to miscarriage, although an increasing number of CNVs were analyzed to be associated with miscarriage. According to bioinformatic analysis as KEGG and GO, the genes performed biological processes like embryonic organ development, vasculogenesis, synapse organization, and histone modification. The following KEGG pathways were accurately related to miscarriages: MAPK signaling pathway, NF-κB signaling pathway, focal adhesion, and HIF-1 signaling pathway. Activating the p38/MAPK signaling pathway inhibits trophoblast proliferation, migration, and epithelial-mesenchymal transition (EMT), resulting in miscarriage [37]. Whereas blocking MAPK/ERK1/2 signaling pathway may inhibit the growth and invasiveness of human trophoblasts [38]. Inhibition of the NF-κB pathway might eliminate the invasiveness of trophoblasts [39]. Moreover, over-activation of NF-κB was observed in early spontaneous miscarriages [40]. HIF-1α/VEGF signaling pathway participates in villous angiogenesis, which is an important event for the development of the placenta or other embryonic organs [41]. A deficiency of HIF-1α may result in fetal heart defects or increase the abortion rate [42]. Our findings were generally in line with previous research, but we did not found genes linked to adherence junction pathway that Wu et al. [21] found to be most enriched in their cohort. This may be due to the differences among samples, more samples and systematic analysis are needed to comprehensively explain the potential mechanism of CNV-related miscarriages.

Further, we selected seven genes (*TP53*, *CTNNB1*, *UBE3A*, *EP300*, *SOX2*, *ATM*, *MECP2*) according to their betweenness with other genes in the PPI network. *TP53* is a vital gene in the pathways of cancers, which functions as a negative regulator to the proliferation of tumors. Summarizing evidence indicates that TP53 is related to miscarriages [43]. *CTNNB1* is an essential participant in the Wnt/β-catenin pathway and a candidate in early embryonic development [44]. *UBE3A* is an imprinted gene related to Angelman syndrome. *EP300*, a member of transcriptional co-activator genes, acts importantly in multiple cellular processes, including embryo development. Inhibition of *Ep300* may induce congenital disabilities and affect gender determination in the embryo [45]. *SOX2* is an essential transcriptional factor for cell differentiation, development, and sexual determination expressed in embryonic stem cells; *SOX2* ablation would cause embryonic lethal after implantation [46]. *ATM* is crucial for DNA repair and apoptosis, and *Atm*^*KD/KD*^ mice died in utero at E9.5 [47]. Zhang et al. [48] discovered that ATM phosphorylates Thr145 and Thr156, two critical resides for the KH domain containing 3like (KHDC3L) which is a new miscarriage risk candidate. *MECP2* plays a crucial role in interpreting epigenetic signatures that command chromatin conformation and regulation of gene transcription. As reported, mutations or increased dosages of the *MECP2* gene may contribute to Rett Syndrome, which is lethal in the male embryo [49]. Moreover, overexpression of *Mecp2* in mice leads to lethal cardiac and skeletal malformations [50]. Misregulation or malfunction of these genes may impair the development or implantation of an embryo, interfering with normal biological processes that might result in miscarriage. The chosen seven genes are somewhat associated with miscarriages, however, the precise relationship between CNVs and miscarriages is yet unclear mainly as the following two reasons. First, most CNVs cover multiple coding genes, thus, it is not easy to pinpoint the causative genes. Second, the changes in gene expression and expression mechanisms are complicated [51]. To clarify the association, basic experiments need to be carried out for functional validation of pathways and genes selected.

According the findings above, it is evident that CNV-Seq is a useful high-resolution technology. In comparison to CMA, which relies on specific probes, CNV-Seq covers the entire genome, resulting in a larger detection region, and it is more cost-effective and convenient. However, it has limitations, as CNV-Seq cannot detect balanced translocation or uniparental disomy (UPD), both of which can be identified by CMA. Considering its high resolution for variants detection, minimal DNA sample requirements, and cost-effectiveness, CNV-Seq may be the preferred choice for clinical use in prenatal diagnosis or miscarriages. It is important to note that while our study indicated that CNVs were detected in most of miscarriages, the origins of variants were not clarified, representing a limitation of this study. In cases where couples are carriers of balanced chromosomal translocation, pregnancies may be lost due to the occurrence of abnormal chromosomes. As chromosomal abnormalities can be inherited from parents or occur de novo, it is essential to find out the origin as a crucial step for future pregnancies.

## Conclusion

To summarize, chromosomal abnormalities were found in most miscarriage cases recruited in this study, particularly in the early abortion group, moreover, 7q36.3 was thought to be a possible CNV for miscarriages. Maternal age was validated as an independent factor of fetal chromosomal abnormalities in miscarriages. Our findings revealed that advanced MA populations have a high risk of aneuploidy, which should be mentioned during eugenic counseling. Still, the relationship between structural chromosomal abnormalities and MA needs further investigation. Clinicians should pay attention to pCNVs and VOUS detected in miscarriages and should be aware of parental sources seeking for CNVs. Bioinformatic analysis indicated participant pathways and central genes of CNVs identified in miscarriages, further functional studies are needed for validation.

### Supplementary Information

**Additional file 1: Table S1.** Pathogenic CNVs (besides aneuploids, and polyploids) detected in 31 abortion samples.

**Additional file 2: Table S2.** List of 21 mosaicisms detected.

**Additional file 3: Table S3.** List of 15 chromosomal disorders overlayed in 56 pCNVs.

## Data Availability

The datasets analysed during the current study are mostly available in the supplemental materials. Due to concerns about individual patient confidentiality, the complete datasets created and analyzed during the current investigation are not publically available, but can be obtained upon reasonable request from the corresponding author.
