# Supplementary material for: Identification of chromosomal abnormalities in miscarriages by CNV-Seq
Source: Mol Cytogenet. 2024 Feb 18;17:4. doi: 10.1186/s13039-024-00671-7 (PMC10875874; doi:10.1186/s13039-024-00671-7)
Supplement: Supplementary file 1 — Additional file 1: Table S1. Pathogenic CNVs (besides aneuploids, and polyploids) detected in 31 abortion samples. [file 13039_2024_671_MOESM1_ESM.docx]

Table S1. Pathogenic CNVs**^a^** (besides aneuploids, and polyploids) detected in 31 abortion samples

| **Case number** | **CNVs** | **Size (Mb)** | **Gene(s)/Syndrome** |
| --- | --- | --- | --- |
| #72 | seq[hg19]del(1)(q21.3);chr1:g.153480000_153920000del | 0.44 | *GATAD2B; S100A6; S100A5; S100A4; S100A3; S100A2; S100A16; S100A14; S100A13; S100A1; CHTOP; SNAPIN; ILF2; NPR1; INTS3; SLC27A3; DENND4B* |
| #169 | seq[hg19]del(5)(p15.33p15.1);chr5:g.40000_17260000del | 17.22 | *PLEKHG4B; LRRC14B; CCDC127; SDHA; PDCD6; AHRR; EXOC3; SLC9A3; CEP72; TPPP; ZDHHC11B; ZDHHC11; BRD9; TRIP13; NKD2; SLC12A7; SLC6A19; SLC6A18; TERT; CLPTM1L; SLC6A3; LPCAT1; MRPL36; NDUFS6; IRX4; IRX2; IRX1; ADAMTS16; ICE1; MED10; UBE2QL1; NSUN2; SRD5A1; TENT4A; ADCY2; C5orf49; FASTKD3; FASTKD3; SEMA5A; TAS2R1; ATPSCKMT; CCT5; CMBL; MARCHF6; ROPN1L; ANKRD33B; DAP; CTNND2; DNAH5; TRIO; OTULINL; ANKH; FBXL7; MARCHF11; ZNF622; RETREG1; MYO10; BASP1*; Cri du chat syndrome [OMIM:123450] |
| #169 | seq[hg19]del(5)(q33.3q35.3);chr5:g.156220000_180700000del | 24.48 | *PPP1R2P3; TIMD4; HAVCR1; HAVCR2; MED7; FAM71B; ITK; CYFIP2; FNDC9; NIPAL4; ADAM19; SOX30; C5orf52; THG1L; LSM11; CLINT1; EBF1; RNF145; UBLCP1; IL12B; ADRA1B; TTC1; PWWP2A; FABP6; CCNJL; C1QTNF2; ZBED8; SLU7; PTTG1; ATP10B; GABRB2; GABRA6; GABRA1; GABRG2; CCNG1; NUDCD2; HMMR; MAT2B; TENM2; WWC1; RARS1; FBLL1; PANK3; SLIT3; SPDL1; DOCK2; INSYN2B; FOXI1; C5orf58; LCP2; KCNIP1; KCNMB1; GABRP; RANBP17; TLX3; NPM1; FGF18; SMIM23; FBXW11; STK10; EFCAB9; UBTD2; SH3PXD2B; NEURL1B; DUSP1; ERGIC1; RPL26L1; ATP6V0E1; CREBRF; BNIP1; NKX2-5; STC2; BOD1; CPEB4; C5orf47; NSG2; MSX2; DRD1; SFXN1; HRH2; CPLX2; THOC3; FAM153B; SIMC1; KIAA1191; ARL10; NOP16; HIGD2A; CLTB; FAF2; RNF44; CDHR2; GPRIN1; SNCB; EIF4E1B; TSPAN17; UNC5A; HK3; UIMC1; ZNF346; FGFR4; NSD1; RAB24; PRELID1; MXD3; LMAN2; RGS14; SLC34A1; PFN3; F12; GRK6; PRR7; DBN1; PDLIM7; DOK3; DDX41; FAM193B; TMED9; B4GALT7; FAM153A; PROP1; N4BP3; RMND5B; NHP2; GMCL1P1; HNRNPAB; PHYKPL; COL23A1; CLK4; ZNF354A; ZNF354B; ZFP2; ZNF454; GRM6; ZNF879; ZNF354C; ADAMTS2; RUFY1; HNRNPH1; C5orf60; CBY3; CANX; MAML1; LTC4S; MGAT4B; SQSTM1; MRNIP; TBC1D9B; RNF130; RASGEF1C; MAPK9; GFPT2; CNOT6; SCGB3A1; FLT4; OR2Y1; MGAT1; ZFP62; BTNL8; BTNL3; BTNL9; OR2V1; OR2V2; TRIM7; TRIM41; RACK1; TRIM52;* Sotos syndrome [OMIM:117550]; |
| #170 | seq[hg19]del(6)(p25.3p25.2);chr6:g.160000_2960000del | 2.80 | *DUSP22; IRF4; EXOC2; HUS1B; FOXQ1; FOXF2;* ***FOXC1****; GMDS; MYLK4; WRNIP1; SERPINB1; SERPINB9; SERPINB9;* |
| #79 | seq[hg19]dup(7)(q35q36.3);chr7:g.144500000_157360000dup | 12.86 | ***TPK1****;* ***CNTNAP2****; C7orf33; CUL1; EZH2; PDIA4; ZNF786; ZNF425; ZNF398; ZNF282; ZNF212; ZNF783; ZNF777; ZNF746; KRBA1; ZNF467; ZNF862; ATP6V0E2; ACTR3C; LRRC61; RARRES2; REPIN1; ZNF775; GIMAP8; GIMAP7; GIMAP4; GIMAP6; GIMAP2; GIMAP1; GIMAP5; TMEM176B; TMEM176A; AOC1;* ***KCNH2****; NOS3; ATG9B; ABCB8; ASIC3; CDK5; SLC4A2; FASTK; TMUB1; AGAP3; GBX1; ASB10; IQCA1L; ABCF2; CHPF2; SMARCD3; NUB1; WDR86; CRYGN; RHEB; PRKAG2; GALNTL5; GALNT11;* ***KMT2C****; XRCC2; ACTR3B;* ***DPP6****; PAXIP1; HTR5A; INSIG1; EN2; CNPY1; RBM33;* ***SHH****; RNF32; LMBR1; NOM1;* ***MNX1****; UBE3C; DNAJB6; PTPRN2*; Polydactyly, preaxial type II [OMIM:174500] |
| #76 | seq[hg19]del(8)(p23.3p21.3);chr8:g.160000_19320000del | 19.16 | 8p23.1 microdeletion syndrome [ORPHA:251071] |
| #76 | seq[hg19]del(8)(p21.3p11.22)(mos);chr8:g.19320000_39380000del | 20.06 | *CSGALNACT1; INTS10; LPL; SLC18A1; ATP6V1B2; LZTS1; GFRA2; DOK2; XPO7; NPM2; FGF17; DMTN; FAM160B2; NUDT18; HR; REEP4; LGI3; SFTPC; BMP1; PHYHIP; POLR3D; PIWIL2; SLC39A14; PPP3CC; SORBS3; PDLIM2; C8orf58; CCAR2; BIN3; EGR3; PEBP4; RHOBTB2; TNFRSF10B; TNFRSF10C; TNFRSF10D; TNFRSF10A; CHMP7; R3HCC1; LOXL2; ENTPD4; SLC25A37; NKX3-1; NKX2-6; STC1; ADAM28; ADAMDEC1; ADAM7; NEFM; NEFL; DOCK5;* ***GNRH1****; KCTD9; CDCA2; EBF2; PPP2R2A; BNIP3L; PNMA2; DPYSL2; ADRA1A; STMN4; TRIM35; PTK2B; CHRNA2; EPHX2; CLU; SCARA3; CCDC25;* ***ESCO2****; PBK; SCARA5; NUGGC; ELP3; PNOC; ZNF395; FBXO16; FZD3; EXTL3; INTS9; HMBOX1; KIF13B; DUSP4; SARAF; LEPROTL1; MBOAT4; DCTN6; RBPMS; GTF2E2; SMIM18; GSR; UBXN8; PPP2CB; TEX15; PURG;* ***WRN****; NRG1; FUT10; MAK16; TTI2; RNF122; DUSP26; UNC5D; KCNU1; ZNF703; ERLIN2; PLPBP; ADGRA2; BRF2; RAB11FIP1; GOT1L1; ADRB3; EIF4EBP1; ASH2L; STAR; LSM1; BAG4; DDHD2; PLPP5; NSD3; LETM2;* ***FGFR1****; TACC1; PLEKHA2; HTRA4; TM2D2;* ***ADAM9****; ADAM32;* |
| #76 | seq[hg19]dup(8)(q21.3q24.3)(mos);chr8:g.91980000_146300000dup | 54.32 | Chromosome 8q22.1 duplication syndrome [OMIM:151200] |
| #108 | seq[hg19]del(8)(p23.3p23.2);chr8:g.160001_5680000del | 5.52 | *ZNF596; FBXO25; TDRP; ERICH1; DLGAP2;* ***CLN8****; ARHGEF10; KBTBD11; MYOM2; CSMD1;* |
| #77 | seq[hg19]dup(11)(q13.4q22.3)(mos);chr11:g.70920000_109860000dup | 38.94 | *SHANK2;* ***DHCR7****; NADSYN1; KRTAP5-7; KRTAP5-8; KRTAP5-9; KRTAP5-10; KRTAP5-11; DEFB108B; RNF121; IL18BP; NUMA1; LAMTOR1; ANAPC15; FOLR3;* ***FOLR1****; FOLR2; INPPL1; PHOX2A; CLPB; PDE2A; ARAP1; STARD10; ATG16L2; FCHSD2; P2RY2; P2RY6; ARHGEF17; RELT; FAM168A; PLEKHB1; RAB6A; MRPL48; COA4; PAAF1; DNAJB13; UCP2; UCP3; C2CD3; PPME1; P4HA3; PGM2L1; KCNE3; LIPT2; POLD3; CHRDL2; RNF169; XRRA1; SPCS2; NEU3; OR2AT4; SLCO2B1; ARRB1; RPS3; KLHL35; GDPD5; SERPINH1; MAP6; MOGAT2; DGAT2; UVRAG; WNT11; THAP12; EMSY; LRRC32; TSKU; ACER3; B3GNT6; CAPN5; OMP; MYO7A; GDPD4; PAK1; AQP11; CLNS1A; RSF1; AAMDC; INTS4; KCTD14; THRSP; NDUFC2; ALG8; KCTD21; USP35; GAB2; NARS2; TENM4; FAM181B; PRCP; DDIAS; RAB30; PCF11; ANKRD42; CCDC90B;* ***DLG2****; TMEM126B; TMEM126A; CREBZF; CCDC89; SYTL2; CCDC83; PICALM; EED; HIKESHI; CCDC81; ME3; PRSS23;* ***FZD4****; TMEM135; RAB38; CTSC; GRM5; TYR; NOX4; TRIM77; TRIM49; TRIM64B; TRIM49D1; TRIM49D2; TRIM64; TRIM49C; UBTFL1; NAALAD2; CHORDC1; FAT3; MTNR1B; SLC36A4; DEUP1; SMCO4; CEP295; TAF1D; C11orf54; MED17; VSTM5; HEPHL1; PANX1; IZUMO1R; GPR83; MRE11; ANKRD49; C11orf97; FUT4; PIWIL4; AMOTL1; CWC15; KDM4D; KDM4E; KDM4F; SRSF8; ENDOD1; SESN3; FAM76B; CEP57;* ***MTMR2****; MAML2; CCDC82; JRKL; CNTN5; ARHGAP42; ARHGAP42; PGR; TRPC6; ANGPTL5; CEP126; CFAP300;* ***YAP1****; BIRC3; BIRC2; TMEM123; MMP7; MMP20; MMP27; MMP8; MMP10; MMP1; MMP3; MMP12; MMP13; DCUN1D5;* ***DYNC2H1****; PDGFD; DDI1; CASP12; CASP4; CASP5; CASP1; CARD16; CARD18; GRIA4; MSANTD4; KBTBD3; AASDHPPT; GUCY1A2; CWF19L2; ALKBH8; ELMOD1; SLN; SLC35F2; RAB39A; CUL5; ACAT1; NPAT;* ***ATM****; C11orf65;* ***POGLUT3****; EXPH5; DDX10; C11orf87;* |
| #77 | seq[hg19]dup(11)(q22.3q23.3)(mos);chr11:g.109860000_116820000dup | 6.96 | *ZC3H12C;* ***RDX****; FDX1; ARHGAP20; C11orf53; COLCA2; POU2AF1; BTG4; HOATZ; LAYN; SIK2; PPP2R1B; ALG9; FDXACB1; C11orf1; CRYAB; HSPB2; C11orf52; DIXDC1; DLAT; PIH1D2; NKAPD1; TIMM8B;* ***SDHD****; IL18; TEX12; BCO2;* ***PTS****; PLET1; NCAM1; TTC12; ANKK1; DRD2; TMPRSS5; ZW10; CLDN25; USP28; HTR3B; HTR3A; ZBTB16; NNMT; C11orf71; RBM7; REXO2; NXPE1; NXPE4; NXPE2; CADM1; BUD13; ZPR1; APOA5; APOA4; APOC3; APOA1; SIK3;* |
| #77 | seq[hg19]dup(11)(q23.3q25);chr11:g.116820000_131500000dup | 14.68 | *SIK3; PAFAH1B2; SIDT2; TAGLN; PCSK7; RNF214; BACE1;* ***CEP164****; DSCAML1; FXYD2; FXYD6l TMPRSS13;* ***IL10RA****; SMIM35; TMPRSS4; SCN4B; SCN2B; JAML; MPZL3; MPZL2;* ***CD3E****;* ***CD3D****;* ***CD3G****; UBE4A; ATP5MG;* ***KMT2A****; TTC36; TMEM25; IFT46;* ***ARCN1****; PHLDB1; TREH; DDX6; CXCR5; BCL9L; UPK2; FOXR1; CCDC84; RPS25; TRAPPC4;* ***SLC37A4****; HYOU1; VPS11;* ***HMBS****; H2AX;* ***DPAGT1****; C2CD2L; HINFP; ABCG4; NLRX1; PDZD3; CCDC153; CBL; MCAM; RNF26; C1QTNF5; USP2; THY1; NECTIN1; TRIM29; OAF; POU2F3; TLCD5; ARHGEF12; GRIK4; TBCEL;* ***TECTA****; SC5D; SORL1; BLID; UBASH3B; CRTAM; JHY; BSX; HSPA8;* ***CLMP****; GRAMD1B; SCN3B; ZNF202; OR6X1; OR6M1; TMEM225; OR8D4; OR4D5; OR6T1; OR10S1; OR10G6; OR10G4; OR10G9; OR10G8; OR10G7; VWA5A; OR10D3; OR8G1; OR8G5; OR8D1; OR8D2; OR8B2; OR8B3; OR8B4; OR8B8; OR8B12; OR8A1; PANX3; TBRG1; SIAE; SPA17; NRGN; VSIG2; ESAM; MSANTD2;* ***ROBO3****; ROBO4; HEPACAM; HEPN1; CCDC15; SLC37A2; TMEM218; PKNOX2; FEZ1; EI24;* ***STT3A****; CHEK1; ACRV1; PATE1; PATE2; PATE3; PATE4;* ***HYLS1****; PUS3; DDX25; CDON; RPUSD4; FAM118B; SRPRA;* ***FOXRED1****; TIRAP; DCPS; ST3GAL4; KIRREL3; ETS1; FLI1;* ***KCNJ1****; KCNJ5; TP53AIP1; ARHGAP32; BARX2; TMEM45B; NFRKB; PRDM10; APLP2;* ***ST14****; ZBTB44; ADAMTS8; ADAMTS15; SNX19; NTM;* |
| #77 | seq[hg19]dup(11)(q25)(mos);chr11:g.131500000_133220000dup | 1.72 | *NTM; OPCML;* |
| #265 | seq[hg19]del(15)(q26.1q26.3);chr15:g.93120000_102400000del | 9.28 | Chromosome 15q26-qter deletion syndrome [OMIM:612626] |
| #266 | seq[hg19]del(18)(q21.33q23);chr18:g.61320001_78020000del | 16.70 | *SERPINB3; SERPINB11;* ***SERPINB7****; SERPINB2; SERPINB10; HMSD; SERPINB8; CDH7; CDH19; DSEL; TMX3; CCDC102B; DOK6; CD226;* ***RTTN****; SOCS6; CBLN2; NETO1; FBXO15; TIMM21;* ***CYB5A****; C18orf63; DIPK1C; CNDP2; CNDP1; ZNF407; ZADH2; TSHZ1; SMIM21; ZNF516; ZNF236; MBP; GALR1; SALL3; ATP9B; NFATC1;* ***CTDP1****; KCNG2; SLC66A2; HSBP1L1; TXNL4A; RBFA; ADNP2; PARD6G;* |
| #267 | seq[hg19]dup(19)(q11q13.43)(mos);chr19:g.27940001_58900000dup | 30.96 | *KMT2B; C19orf12; SLC7A9; PEPD; COX6B1; NPHS1; TYROBP; SDHAF1; SYNE4; WDR62; RPS19; ERF; CIC; APOC2; ERCC2; ERCC1; CCDC8; LMTK3; POLD1; SHANK1; ETFB; GP6; TNNT1; TNNI3;* |
| #168 | seq[hg19]del(Y)(p11.31);chrY:g.2640001_2860000del | 0.22 | *SRY; RPS4Y1; ZFY;* |
| #109 | seq[hg19]del(15)(q11.2q15.2);chr15:g.22760000_43520000del | 20.76 | Prader-Willi syndrome [OMIM:176270]; Angelman syndrome [OMIM:105830]; Chromosome 15q13.3 deletion syndrome [OMIM:612001] |
| #73 | seq[hg19]dup(6)(p25.3q23.3);chr6:g.160000_136180000dup | 136.02 | *FOXC1; SERPINB6; FARS2; F13A1; DSP; NEDD9; ALDH5A1; HFE; CYP21A2; SYNGAP1; FANCE; PPP2R5D; POLH; VEGFA; AARS2; MMUT; TFAP2B; PHF3; EYS; RIMS1; KHDC3L; MTO1; SLC17A5; PHIP; LCA5; BCKDHB; PGM3; RARS2; SIM1; GRIK2; PDSS2; SOBP; OSTM1; ZBTB24; CCN6; DSE; RSPH4A; RFX6; NUS1; TRDN; LAMA2; ARG1; ENPP1; EYA4; AHI1;* |
| #73 | seq[hg19]dup(3)(p26.3p14.2);chr3:g.60000_60520000dup | 60.46 | *SETD5; BRPF1; VHL; SLC6A1; MLH1; SCN5A; CTNNB1; SETD2; BAP1; SUMF1; ITPR1; CIDEC; FANCD2; TSEN2; WNT7A; XPC; COLQ; BTD; RPL15; NGLY1; GLB1; CRTAP; FYCO1; TDGF1; COL7A1;* |
| #74 | seq[hg19]del(7)(q36.3);chr7:g.155140000_159138663del | 4.00 | *EN2; CNPY1; RBM33;* ***SHH****; RNF32;* ***LMBR1****; NOM1;* ***MNX1****; UBE3C; DNAJB6; PTPRN2; NCAPG2; ESYT2;* ***DYNC2I1****; VIPR2;* |
| #74 | seq[hg19]del(18)(q22.2q23);chr18:g.68360000_78020000del | 9.66 | *CBLN2; NETO1; FBXO15; TIMM21;* ***CYB5A****; C18orf63; DIPK1C; CNDP2; CNDP1; ZNF407; ZADH2; TSHZ1; SMIM21; ZNF516; ZNF236; MBP; GALR1; SALL3; ATP9B; NFATC1;* ***CTDP1****; KCNG2; SLC66A2; HSBP1L1; TXNL4A; RBFA; ADNP2; PARD6G;* |
| #74 | seq[hg19]del(15)(q25.2q26.3);chr15:g.83920000_102400000del | 20.76 | Chromosome 15q26-qter deletion syndrome [OMIM:612626] |
| #75 | seq[hg19]dup(8)(q24.11q24.3)(mos);chr8:g.117920000_146300000dup | 28.38 | *AARD; SLC30A8; MED30;* ***EXT1****; SAMD12;* ***TNFRSF11B****; COLEC10; MAL2; CCN3; ENPP2;* ***TAF2****; DSCC1; DEPTOR; COL14A1; MRPL13; MTBP; SNTB1; HAS2; ZHX2; DERL1; TBC1D31; FAM83A; C8orf76; ZHX1; ATAD2; NTAQ1; FBXO32; KLHL38; ANXA13; FAM91A1; FER1L6; TMEM65; TRMT12; RNF139; TATDN1; NDUFB9; MTSS1; ZNF572; SQLE; WASHC5; NSMCE2; TRIB1; LRATD2; POU5F1B; MYC; GSDMC; CYRIB; ASAP1; ADCY8; EFR3A; OC90; HHLA1; KCNQ3; LRRC6; TMEM71; PHF20L1; TG; SLA; CCN4;* ***NDRG1****; ST3GAL1; ZFAT; KHDRBS3; FAM135B; COL22A1; KCNK9;* ***TRAPPC9****; CHRAC1; AGO2; PTK2; DENND3; SLC45A4; GPR20; PTP4A3; MROH5; TSNARE1; ADGRB1; ARC; JRK; PSCA; LY6K; THEM6;* ***SLURP1****; LYPD2; LYNX1; LY6D; GML; CYP11B1; CYP11B2; LY6E; LY6H;* ***GPIHBP1****; ZFP41; GLI4; ZNF696; TOP1MT; RHPN1; MAFA; ZC3H3; GSDMD; MROH6; NAPRT; EEF1D; TIGD5; PYCR3; GFUS; ZNF623; ZNF707; CCDC166; MAPK15; FAM83H; SCRIB; PUF60; NRBP2; EPPK1; PLEC; PARP10; GRINA; SPATC1; OPLAH; EXOSC4; GPAA1; CYC1; SHARPIN; MAF1; WDR97; HGH1; MROH1; BOP1; SCX; HGH1; SCX; HSF1; DGAT1; SCRT1; TMEM249; FBXL6; SLC52A2; ADCK5; CPSF1; SLC39A4; VPS28; TONSL; CYHR1; KIFC2; FOXH1; PPP1R16A; GPT; MFSD3; RECQL4; LRRC14; LRRC24; C8orf82; ARHGAP39; ZNF251; ZNF34; RPL8; ZNF517; ZNF7; COMMD5; ZNF250; ZNF16; C8orf33;* |
| #75 | seq[hg19]dup(9)(q21.32q34.3);chr9:g.86500000_141020000dup | 54.52 | PHF2; FBP1; FANCC; ERCC6L2; PTCH1; HSD17B3; XPA; FOXE1; ANKS6; GALNT12; TGFBR1; ALG2; ZNF462; ALAD; WHRN; ASTN2; TRIM32; CDK5RAP2; NR5A1; STXBP1; DNM1; SURF1; ADAMTS13; ADAMTSL2; DBH; COL5A1; LHX3; INPP5E; AGPAT2; TPRN; EHMT1; |
| #75 | seq[hg19]del(11)(q24.1q25);chr11:g.122380001_134960000del | 12.58 | *UBASH3B; CRTAM; JHY; BSX; HSPA8;* ***CLMP****; GRAMD1B; SCN3B; ZNF202; OR6X1; OR6M1; TMEM225; OR8D4; OR4D5; OR6T1; OR10S1; OR10G6; OR10G4; OR10G9; OR10G8; OR10G7; VWA5A; OR10D3; OR8G1; OR8G5; OR8D1; OR8D2; OR8B2; OR8B3; OR8B4; OR8B8; OR8B12; OR8A1; PANX3; TBRG1; SIAE; SPA17; NRGN; VSIG2; ESAM; MSANTD2;* ***ROBO3****; ROBO4; HEPACAM; HEPN1; CCDC15; SLC37A2; TMEM218; PKNOX2; FEZ1; EI24;* ***STT3A****; CHEK1; ACRV1; PATE1; PATE2; PATE3; PATE4;* ***HYLS1****; PUS3; DDX25; CDON; RPUSD4; FAM118B; SRPRA;* ***FOXRED1****; TIRAP; DCPS; ST3GAL4; KIRREL3; ETS1; FLI1;* ***KCNJ1****; KCNJ5; TP53AIP1; ARHGAP32; BARX2; TMEM45B; NFRKB; PRDM10; APLP2;* ***ST14****; ZBTB44; ADAMTS8; ADAMTS15; SNX19; NTM; OPCML; SPATA19; IGSF9B; IGSF9B;* ***JAM3****; NCAPD3; VPS26B; THYN1;* ***ACAD8****; GLB1L3; GLB1L2; B3GAT1;* |
| #75 | seq[hg19]del(18)(p11.32p11.21);chr18:g.120001_11440000del | 11.32 | Chromosome 18p deletion syndrome [OMIM:146390] |
| #99 | seq[hg19]dup(3)(q25.32q29);chr3:g.157060001_197840000dup | 40.78 | Chromosome 3q29 microduplication syndrome [OMIM:611936] |
| #101 | seq[hg19]del(4)(p16.1p15.1)(mos);chr4:g.7160001_32520000del | 25.36 | Wolf-Hirschhorn syndrome [OMIM:194190] |
| #103 | seq[hg19]del(4)(p16.3p16.1);chr4:g.40001_7160000del | 7.12 | Wolf-Hirschhorn syndrome [OMIM:194190] |
| #104 | seq[hg19]del(5)(p15.33p15.32);chr5:g.20001_4660000del | 4.64 | *PLEKHG4B; LRRC14B; CCDC127; SDHA; PDCD6; AHRR; EXOC3; SLC9A3; CEP72; TPPP; ZDHHC11B; ZDHHC11; BRD9; TRIP13; NKD2; SLC12A7; SLC6A19; SLC6A18; TERT; CLPTM1L;* ***SLC6A3****; LPCAT1; MRPL36;* ***NDUFS6****; IRX4; IRX2; IRX1;* |
| #104 | seq[hg19]del(7)(q32.3q36.3);chr7:g.131800001_159138663del | 27.34 | *PLXNA4; CHCHD3; EXOC4; LRGUK; SLC35B4; AKR1B1; AKR1B10; AKR1B15; BPGM; CALD1; AGBL3; CYREN; TMEM140; WDR91; STRA8; CNOT4; NUP205; STMP1; SLC13A4; FAM180A; MTPN; CHRM2; PTN; DGKI; CREB3L2; AKR1D1; TRIM24; SVOPL;* ***ATP6V0A4****; TMEM213; KIAA1549; ZC3HAV1L; ZC3HAV1; TTC26;* ***UBN2****; LUC7L2; FMC1; KLRG2; CLEC2L; HIPK2; TBXAS1; PARP12; KDM7A; SLC37A3; RAB19; MKRN1; DENND2A; ADCK2; NDUFB2;* ***BRAF****; MRPS33; TMEM178B;* ***AGK****; DENND11; WEE2; SSBP1; TAS2R3; TAS2R4; TAS2R5; PRSS37; OR9A4; CLEC5A; TAS2R38; MGAM; MGAM2; PRSS58; TRB; PRSS1; EPHB6; TRPV6; TRPV5; LLCFC1; KEL; OR9A2; OR6V1; PIP; TAS2R39; TAS2R40; GSTK1; TMEM139; CASP2; CLCN1; FAM131B; ZYX; EPHA1; TAS2R60; TAS2R41; OR10AC1; CTAGE15; TCAF2; CTAGE6; TCAF1; OR2F2; OR2F1; OR6B1; OR2A5; OR2A25; OR2A12; OR2A2; OR2A14; CTAGE4; ARHGEF35; OR2A42; OR2A7; CTAGE8; OR2A1; ARHGEF5; NOBOX;* ***TPK1****;* ***CNTNAP2****; C7orf33; CUL1; EZH2; PDIA4; ZNF786; ZNF425; ZNF398; ZNF282; ZNF212; ZNF783; ZNF777; ZNF746; KRBA1; ZNF467; ZNF862; ATP6V0E2; ACTR3C; LRRC61; RARRES2; REPIN1; ZNF775; GIMAP8; GIMAP7; GIMAP4; GIMAP6; GIMAP2; GIMAP1; GIMAP5; TMEM176B; TMEM176A; AOC1;* ***KCNH2****; NOS3; ATG9B; ABCB8; ASIC3; CDK5; SLC4A2; FASTK; TMUB1; AGAP3; GBX1; ASB10; IQCA1L; ABCF2; CHPF2; SMARCD3; NUB1; WDR86; CRYGN; RHEB; PRKAG2; GALNTL5; GALNT11;* ***KMT2C****; XRCC2; ACTR3B;* ***DPP6****; PAXIP1; HTR5A; INSIG1; EN2; CNPY1; RBM33; SHH; RNF32; LMBR1; NOM1; MNX1; UBE3C; DNAJB6; PTPRN2; NCAPG2; ESYT2; DYNC2I1; VIPR2;* |
| #106 | seq[hg19]dup(11)(p15.5p15.4);chr11:g.180001_7960000dup | 7.78 | *SCGB1C1; ODF3; BET1L; RIC8A; SIRT3; PSMD13; NLRP6; PGGHG; IFITM5; IFITM2; IFITM1; IFITM3; B4GALNT4; PKP3; SIGIRR; ANO9; PTDSS2; RNH1; HRAS; LRRC56; LMNTD2; RASSF7; PHRF1; IRF7; CDHR5; SCT; DRD4; DEAF1; TMEM80; EPS8L2; TALDO1; GATD1; CEND1; SLC25A22; PANO1; PIDD1; RPLP2; PNPLA2; CRACR2B; CD151; POLR2L; TSPAN4; CHID1; AP2A2; MUC6; MUC2; MUC5AC; MUC5B; TOLLIP; BRSK2; MOB2; DUSP8; KRTAP5-1; KRTAP5-2; KRTAP5-3; KRTAP5-4; KRTAP5-5; KRTAP5-6; IFITM10; CTSD; SYT8; TNNI2; LSP1; TNNT3; MRPL23; IGF2; INS; TH; ASCL2; C11orf21; TSPAN32; CD81; TSSC4; TRPM5;* ***KCNQ1****;* ***CDKN1C****; SLC22A18; PHLDA2; NAP1L4; CARS1; OSBPL5; MRGPRG; MRGPRE; ZNF195; ART5; ART1; CHRNA10; NUP98; PGAP2; RHOG; STIM1; RRM1; SSU72P5; SSU72P2; SSU72P4; SSU72P3; SSU72P7; OR52B4; TRIM21; OR52K2; OR52K1; OR52M1; OR52I2; OR52I1; TRIM68; OR51D1; OR51E1; OR51E2; OR51F1; OR52R1; OR51F2; OR51S1; OR51H1; OR51T1; OR51A7; OR51G2; OR51G1; OR51A4; OR51A2; MMP26; OR51L1; OR52J3; OR52E2; OR52E1; OR52A5; OR52A1; OR51V1; HBB; HBD; HBG1; HBG2; HBE1; OR51B4; OR51B2; OR51B5; OR51B6; OR51M1; OR51J1; OR51Q1; OR51I1; OR51I2; OR52D1; UBQLN3; UBQLNL; OR52H1; OR52B6; TRIM6; TRIM34; TRIM5; TRIM22; OR52P1P; OR56B1; OR52N4; OR52N5; OR52N1; OR52N2; OR52E6; OR52E8; OR52E4; OR52E5; OR56A3; OR56A5; OR52L1; OR56A4; OR56A1; OR56B4; OR52B2; OR52W1; C11orf42; FAM160A2; CNGA4; CCKBR; CAVIN3; SMPD1; APBB1; HPX; TRIM3; ARFIP2; TIMM10B; DNHD1; RRP8; ILK; TAF10; TPP1;* ***DCHS1****; MRPL17; OR2AG2; OR2AG1; OR6A2; OR10A5; OR10A2; OR10A4; OR2D2; OR2D3; ZNF215; ZNF214; NLRP14; RBMXL2; SYT9; OLFML1; PPFIBP2; CYB5R2; OVCH2; OR5P2; OR5P3; OR10A6;* |
| #106 | seq[hg19]del(8)(p23.3p12);chr8:g.160000_35840000del | 35.68 | *ZNF596; FBXO25; TDRP; ERICH1; DLGAP2;* ***CLN8****; ARHGEF10; KBTBD11; MYOM2;* ***CSMD1****;* ***MCPH1****; ANGPT2; AGPAT5; XKR5; DEFB1; DEFA6; DEFA4; DEFA1; DEFA1B; DEFA3; DEFA5; FAM90A15P; FAM90A3P; FAM90A13P; FAM90A5P; FAM90A20P; DEFB109P1B; USP17L1; USP17L4; ZNF705G; DEFB4B; DEFB103B; SPAG11B; DEFB104B; DEFB106B; DEFB105B; DEFB107B; PRR23D1; FAM90A7P; FAM90A22P; FAM90A23P; FAM90A14P; FAM90A18P; FAM90A16P; FAM90A8P; FAM90A17P; FAM90A19P; FAM90A9P; FAM90A10P; PRR23D2; DEFB107A; DEFB105A; DEFB106A; DEFB104A; SPAG11A; DEFB103A; DEFB4A; ZNF705B; USP17L8; USP17L3; FAM90A11P; FAM90A24P; FAM90A12P; PRAG1; CLDN23; MFHAS1; ERI1; PPP1R3B; TNKS; MSRA; PRSS55; RP1L1; C8orf74; SOX7; PINX1; XKR6; MTMR9; SLC35G5; FAM167A; BLK;* ***GATA4****; NEIL2; FDFT1; CTSB; DEFB136; DEFB135; DEFB134; ZNF705D; USP17L7; USP17L2; FAM86B1; DEFB130A; FAM86B2; LONRF1; TRMT9B; DLC1; C8orf48; SGCZ;* ***TUSC3****; MSR1; FGF20; MICU3; ZDHHC2; CNOT7;* ***VPS37A****; MTMR7; SLC7A2; PDGFRL; MTUS1; FGL1; PCM1;* ***ASAH1****; NAT1; NAT2; PSD3; SH2D4A; CSGALNACT1; INTS10; LPL; SLC18A1; ATP6V1B2; LZTS1; GFRA2; DOK2; XPO7; NPM2; FGF17; DMTN; FAM160B2; NUDT18; HR; REEP4; LGI3; SFTPC; BMP1; PHYHIP; POLR3D; PIWIL2; SLC39A14; PPP3CC; SORBS3; PDLIM2; C8orf58; CCAR2; BIN3; EGR3; PEBP4; RHOBTB2; TNFRSF10B; TNFRSF10C; TNFRSF10D; TNFRSF10A; CHMP7; R3HCC1; LOXL2; ENTPD4; SLC25A37; NKX3-1; NKX2-6; STC1; ADAM28; ADAMDEC1; ADAM7; NEFM; NEFL; DOCK5;* ***GNRH1****; KCTD9; CDCA2; EBF2; PPP2R2A; BNIP3L; PNMA2; DPYSL2; ADRA1A; STMN4; TRIM35; PTK2B; CHRNA2; EPHX2; CLU; SCARA3; CCDC25;* ***ESCO2****; PBK; SCARA5; NUGGC; ELP3; PNOC; ZNF395; FBXO16; FZD3; EXTL3; INTS9; HMBOX1; KIF13B; DUSP4; SARAF; LEPROTL1; MBOAT4; DCTN6; RBPMS; GTF2E2; SMIM18; GSR; UBXN8; PPP2CB; TEX15; PURG;* ***WRN****; NRG1; FUT10; MAK16; TTI2; RNF122; DUSP26; UNC5D;* |
| #106 | seq[hg19]del(7)(q32.3q36.3);chr7:g.131800001_159138663del | 27.34 | PLXNA4; CHCHD3; EXOC4; LRGUK; SLC35B4; AKR1B1; AKR1B10; AKR1B15; BPGM; CALD1; AGBL3; CYREN; TMEM140; WDR91; STRA8; CNOT4; NUP205; STMP1; SLC13A4; FAM180A; MTPN; CHRM2; PTN; DGKI; CREB3L2; AKR1D1; TRIM24; SVOPL; ATP6V0A4; TMEM213; KIAA1549; ZC3HAV1L; ZC3HAV1; TTC26; UBN2; LUC7L2; FMC1; KLRG2; CLEC2L; HIPK2; TBXAS1; PARP12; KDM7A; SLC37A3; RAB19; MKRN1; DENND2A; ADCK2; NDUFB2; BRAF; MRPS33; TMEM178B; AGK; DENND11; WEE2; SSBP1; TAS2R3; TAS2R4; TAS2R5; PRSS37; OR9A4; CLEC5A; TAS2R38; MGAM; MGAM2; PRSS58; TRB; PRSS1; EPHB6; TRPV6; TRPV5; LLCFC1; KEL; OR9A2; OR6V1; PIP; TAS2R39; TAS2R40; GSTK1; TMEM139; CASP2; CLCN1; FAM131B; ZYX; EPHA1; TAS2R60; TAS2R41; OR10AC1; CTAGE15; TCAF2; CTAGE6; TCAF1; OR2F2; OR2F1; OR6B1; OR2A5; OR2A25; OR2A12; OR2A2; OR2A14; CTAGE4; ARHGEF35; OR2A42; OR2A7; CTAGE8; OR2A1; ARHGEF5; NOBOX; TPK1; CNTNAP2; C7orf33; CUL1; EZH2; PDIA4; ZNF786; ZNF425; ZNF398; ZNF282; ZNF212; ZNF783; ZNF777; ZNF746; KRBA1; ZNF467; ZNF862; ATP6V0E2; ACTR3C; LRRC61; RARRES2; REPIN1; ZNF775; GIMAP8; GIMAP7; GIMAP4; GIMAP6; GIMAP2; GIMAP1; GIMAP5; TMEM176B; TMEM176A; AOC1; KCNH2; NOS3; ATG9B; ABCB8; ASIC3; CDK5; SLC4A2; FASTK; TMUB1; AGAP3; GBX1; ASB10; IQCA1L; ABCF2; CHPF2; SMARCD3; NUB1; WDR86; CRYGN; RHEB; PRKAG2; GALNTL5; GALNT11; KMT2C; XRCC2; ACTR3B; DPP6; PAXIP1; HTR5A; INSIG1; EN2; CNPY1; RBM33; SHH; RNF32; LMBR1; NOM1; MNX1; UBE3C; DNAJB6; PTPRN2; NCAPG2; ESYT2; DYNC2I1; VIPR2; |
| #107 | seq[hg19]del(8)(p12p11.21)(mos);chr8:g.35840000_39880000del | 4.04 | *KCNU1; ZNF703; ERLIN2; PLPBP; ADGRA2; BRF2; RAB11FIP1; GOT1L1; ADRB3; EIF4EBP1; ASH2L; STAR; LSM1; BAG4; DDHD2; PLPP5; NSD3; LETM2;* ***FGFR1****; TACC1; PLEKHA2; HTRA4; TM2D2;* ***ADAM9****; ADAM32; ADAM18; ADAM2; IDO1; IDO2;* |
| #107 | seq[hg19]dup(8)(q11.1q24.3)(mos);chr8:g.47840001_146300000dup | 98.46 | Chromosome 8q22.1 duplication syndrome [OMIM:151200] |
| #107 | seq[hg19]del(1)(p36.33p36.12);chr1:g.840000_21600000del | 20.76 | Chromosome 1p36 deletion syndrome [OMIM:607872]; CAMTA1 [OMIM:611501] |
| #166 | seq[hg19]dup(5)(q35.2q35.3);chr5:g.172900000_180700000dup | 7.80 | Sotos syndrome [OMIM:117550] |
| #166 | seq[hg19]del(6)(q24.3q27);chr6:g.145940000_170920000del | 24.98 | ARID1B [OMIM:614556]; DLL1 [OMIM:606582] |
| #171 | seq[hg19]dup(7)(q21.3q36.3);chr7:g.94840000_159138663dup | 64.30 | Polydactyly, preaxial type II [OMIM:174500] |
| #171 | seq[hg19]del(X)(p22.33p11.22);chrX:g.2700000_51960000del | 49.26 | Ichthyosis, X-linked [OMIM:308100]; Chromosome Xp21 deletion syndrome [OMIM:300679]; Chromosome Xp11.3 deletion syndrome [OMIM:300578] |
| #172 | seq[hg19]del(X)(p11.21q28)(mos);chrX:g.56740000_154940000del | 98.20 | Moyamoya disease 4 [OMIM:300845]; AMME complex [OMIM:300194]; Chromosome Xq21 deletion syndrome [OMIM:303110] |
| #172 | seq[hg19]dup(8)(q12.1);chr8:g.57000000_57200000dup | 0.20 | *MOS; PLAG1; CHCHD7;* |
| #172 | seq[hg19]dup(8)(q12.1q24.3);chr8:g.57200000_146300000dup | 89.10 | Chromosome 8q22.1 duplication syndrome [OMIM:151200] |
| #172 | seq[hg19]dup(X)(p11.22p11.21)(mos);chrX:g.51960000_56740000dup | 4.78 | *XAGE2; XAGE1B; XAGE1A; SSX7; SSX2; SSX2B; SPANXN5; XAGE5; XAGE3; FAM156B; FAM156A; GPR173; TSPYL2; KANTR;* ***KDM5C****;* ***IQSEC2****;* ***SMC1A****; RIBC1; HSD17B10; HUWE1;* ***PHF8****; FAM120C; WNK3; TSR2;* ***FGD1****; GNL3L; ITIH6; MAGED2; TRO; PFKFB1; APEX2; ALAS2; PAGE2B; PAGE2; FAM104B; PAGE5; PAGE3; MAGEH1; USP51; FOXR2; RRAGB; KLF8; UBQLN2;* |
| #172 | seq[hg19]dup(1)(q25.3q44)(mos);chr1:g.182740000_249220000dup | 66.48 | *LAMC2; NCF2; PRG4; CDC73; F13B; ASPM; UBE2T; KDM5B; KISS1; CNTN2; IRF6; SYT14; USH2A; SLC30A10; RAB3GAP2; WDR26; LBR; AGT; GNPAT; TBCE; B3GALNT2; LYST; MTR; FH; SDCCAG8; AKT3; ZBTB18; COX20;* |
| #173 | seq[hg19]dup(8)(p11.22q24.3)(mos)chr8:g.39380000_146300000dup | 106.92 | Chromosome 8q22.1 duplication syndrome [OMIM:151200] |
| #173 | seq[hg19]del(X)(p11.23);chrX:g.48620000_48900000del | 0.28 | SLC35A2[OMIM:314375]; PQBP1 [OMIM:300463] |
| #263 | seq[hg19]del(17)(p13.3p12);chr17:g.1_13340000del | 13.34 | Miller-Dieker lissencephaly syndrome [OMIM:247200]; Chromosome 17p13.1 deletion syndrome [OMIM:613776] |
| #264 | seq[hg19]dup(10)(p15.3p12.31);chr10:g.120001_20320000dup | 20.20 | ***ZMYND11****; DIP2C; LARP4B; GTPBP4; IDI2; IDI1; WDR37; ADARB2; PFKP; PITRM1; KLF6; AKR1E2; AKR1C1; AKR1C2; AKR1C3; AKR1C8P; AKR1C4; UCN3; TUBAL3; NET1; CALML5; CALML3; ASB13; TASOR2; GDI2; ANKRD16; FBH1; IL15RA;* ***IL2RA****; RBM17; PFKFB3; PRKCQ; SFMBT2; ITIH5; ITIH2; KIN; ATP5F1C; TAF3;* ***GATA3****; CELF2; USP6NL; ECHDC3; PROSER2; UPF2; DHTKD1; SEC61A2; NUDT5; CDC123; CAMK1D; CCDC3; OPTN; MCM10; UCMA; PHYH; SEPHS1; BEND7; PRPF18; FRMD4A; FAM107B; CDNF; HSPA14; SUV39H2;* ***DCLRE1C****; MEIG1; OLAH; ACBD7; RPP38; NMT2; FAM171A1; ITGA8; MINDY3; PTER; C1QL3; RSU1;* ***CUBN****; TRDMT1; VIM; ST8SIA6; HACD1; STAM; TMEM236; MRC1; SLC39A12; CACNB2; NSUN6; ARL5B; MALRD1; PLXDC2;* |
| #264 | seq[hg19]dup(11)(p15.5p15.4);chr11:g.180001_7700000dup | 7.78 | *SCGB1C1; ODF3; BET1L; RIC8A; SIRT3; PSMD13; NLRP6; PGGHG; IFITM5; IFITM2; IFITM1; IFITM3; B4GALNT4; PKP3; SIGIRR; ANO9; PTDSS2; RNH1; HRAS; LRRC56; LMNTD2; RASSF7; PHRF1; IRF7; CDHR5; SCT; DRD4; DEAF1; TMEM80; EPS8L2; TALDO1; GATD1; CEND1; SLC25A22; PANO1; PIDD1; RPLP2; PNPLA2; CRACR2B; CD151; POLR2L; TSPAN4; CHID1; AP2A2; MUC6; MUC2; MUC5AC; MUC5B; TOLLIP; BRSK2; MOB2; DUSP8; KRTAP5-1; KRTAP5-2; KRTAP5-3; KRTAP5-4; KRTAP5-5; KRTAP5-6; IFITM10; CTSD; SYT8; TNNI2; LSP1; TNNT3; MRPL23; IGF2; INS; TH; ASCL2; C11orf21; TSPAN32; CD81; TSSC4; TRPM5;* ***KCNQ1****;* ***CDKN1C****; SLC22A18; PHLDA2; NAP1L4; CARS1; OSBPL5; MRGPRG; MRGPRE; ZNF195; ART5; ART1; CHRNA10; NUP98; PGAP2; RHOG; STIM1; RRM1; SSU72P5; SSU72P2; SSU72P4; SSU72P3; SSU72P7; OR52B4; TRIM21; OR52K2; OR52K1; OR52M1; OR52I2; OR52I1; TRIM68; OR51D1; OR51E1; OR51E2; OR51F1; OR52R1; OR51F2; OR51S1; OR51H1; OR51T1; OR51A7; OR51G2; OR51G1; OR51A4; OR51A2; MMP26; OR51L1; OR52J3; OR52E2; OR52E1; OR52A5; OR52A1; OR51V1; HBB; HBD; HBG1; HBG2; HBE1; OR51B4; OR51B2; OR51B5; OR51B6; OR51M1; OR51J1; OR51Q1; OR51I1; OR51I2; OR52D1; UBQLN3; UBQLNL; OR52H1; OR52B6; TRIM6; TRIM34; TRIM5; TRIM22; OR52P1P; OR56B1; OR52N4; OR52N5; OR52N1; OR52N2; OR52E6; OR52E8; OR52E4; OR52E5; OR56A3; OR56A5; OR52L1; OR56A4; OR56A1; OR56B4; OR52B2; OR52W1; C11orf42; FAM160A2; CNGA4; CCKBR; CAVIN3; SMPD1; APBB1; HPX; TRIM3; ARFIP2; TIMM10B; DNHD1; RRP8; ILK; TAF10; TPP1;* ***DCHS1****; MRPL17; OR2AG2; OR2AG1; OR6A2; OR10A5; OR10A2; OR10A4; OR2D2; OR2D3; ZNF215; ZNF214; NLRP14; RBMXL2; SYT9; OLFML1; PPFIBP2; CYB5R2;* |
| #337 | seq[hg19]del(13)(q21.32q34)(mos);chr13:g.65940001_115100000del | 49.16 | *PCDH9; KLHL1; DACH1; MZT1; BORA; DIS3; PIBF1; KLF5; KLF12; TBC1D4; COMMD6; UCHL3; LMO7; KCTD12; ACOD1; CLN5; FBXL3; MYCBP2; SCEL; SLAIN1;* ***EDNRB****; POU4F1; OBI1; RBM26; NDFIP2; SPRY2; SLITRK1; SLITRK6; SLITRK5; GPC5;* ***GPC6****; DCT; TGDS; GPR180; SOX21; ABCC4; CLDN10; DZIP1; DNAJC3; UGGT2; HS6ST3; OXGR1; MBNL2; RAP2A; IPO5; FARP1; RNF113B; STK24; SLC15A1; DOCK9; UBAC2; GPR18; GPR183; TM9SF2; CLYBL; ZIC5;* ***ZIC2****;* ***PCCA****; GGACT; TMTC4; NALCN; ITGBL1; FGF14; TPP2; METTL21C; CCDC168; TEX30; POGLUT2; BIVM;* ***ERCC5****; SLC10A2; DAOA; EFNB2; ARGLU1; FAM155A;* ***LIG4;*** *ABHD13; TNFSF13B; MYO16; IRS2; COL4A1; COL4A2; RAB20; NAXD; CARS2; ING1; ANKRD10; ARHGEF7; TEX29; SOX1; SPACA7; TUBGCP3; ATP11A; MCF2L;* ***F7****;* ***F10****; PROZ; PCID2; CUL4A; LAMP1; GRTP1; ADPRHL1; DCUN1D2; TMCO3; TFDP1; ATP4B; GRK1; TMEM255B; GAS6; RASA3; CDC16; UPF3A;* ***CHAMP1****;* |
| #214 | seq[hg19]del(22)(q13.2);chr22:g.41440000_41700000del | 0.26 | ***EP300****; L3MBTL2; CHADL; RANGAP1; ZC3H7B;* |
| #262 | seq[hg19]del(17)(p12);chr17:g.14100000_15420000del | 1.32 | ***COX10****; CDRT15; HS3ST3B1; PMP22; TEKT3; CDRT4; TVP23C;* |
| #98 | seq[hg19]del(4)(p16.3p15.33);chr4:g.40000_13920000del | 13.88 | Wolf-Hirschhorn syndrome [OMIM:194190] |

**^a^** International System for Human Cytogenomic Nomenclature (ISCN) 2020 is used for CNV description
