# Supplementary material for: Identification of chromosomal abnormalities in miscarriages by CNV-Seq
Source: Mol Cytogenet. 2024 Feb 18;17:4. doi: 10.1186/s13039-024-00671-7 (PMC10875874; doi:10.1186/s13039-024-00671-7)
Supplement: Supplementary file 2 — Additional file 2: Table S2. List of 21 mosaicisms detected. [file 13039_2024_671_MOESM2_ESM.docx]

Table S2 List of 21 mosaicisms detected

| **Case number** | **Mosaicisms** |
| --- | --- |
| #83 | 47,XN,+14[25%]/46,XN[75%] |
| #97 | 47,XNN[15%]/46,XN[85%] |
| #98 | 47,XN,+16[20%]/46,XN[80%] |
| #175 | 47,XXX[80%]/46,XXX,-21[20%] |
| #248 | 47,XN,+22[70%]/46,XN[30%] |
| #255 | 47,XN,+7[75%]/48,XN,+7,+13[25%] |
| #256 | 47,XN,+7[60%]/46,XN[40%] |
| #257 | 47,XN,+16[80%]/49,XN,+7,+12,+16[20%] |
| #332 | 45,X[10%]/46,XN[90%] |
| #333 | 45,X[20%]/46,XY[80%] |
| #334 | 45,X[90%]/46,XY[10%] |
| #335 | 47,XN,+16[50%]/46,XN[50%] |
| #336 | 47,XN,+20[40%]/46,XN[60%] |
| #337 | 47,XN,+4[10%]/46,XN[90%] |
| #338 | 47,XN,+8[70%]/46,XN[30%] |
| #339 | 48,XN,+16,+22[60%]/47,XN,+16[40%] |
| #340 | 48,XN,+2,+16[60%]/47,XN,+2[40%] |
| #341 | 48,XN,+4,+7[55%]/46,XN[45%] |
| #348 | 70,XNN,+3[60%]/69,XNN[40%] |
| #393 | 69,XNN[50%]/70,XNN,+2[50%]; |
| #394 | 69,XNN[65%]/70,XNN,+14[35%] |
