# Supplementary material for: Identification of chromosomal abnormalities in miscarriages by CNV-Seq
Source: Mol Cytogenet. 2024 Feb 18;17:4. doi: 10.1186/s13039-024-00671-7 (PMC10875874; doi:10.1186/s13039-024-00671-7)
Supplement: Supplementary file 3 — Additional file 3: Table S3. List of 15 chromosomal disorders overlayed in 56 pCNVs. [file 13039_2024_671_MOESM3_ESM.docx]

Table S3 List of 15 chromosomal disorders overlayed in 56 pCNVs

| **Chromosomal disorders** | **Number (frequency)** | **Case number** |
| --- | --- | --- |
| Chromosome 8q22.1 duplication syndrome (OMIM: #151200), | 4 (26.32%) | #76, #107, #172, #173 |
| Wolf-Hirschhorn syndrome (OMIM: #194190) | 2 (10.53%) | #101, #98 |
| Cri du chat syndrome (OMIM: #123450) | 1 (5.26%) | #169 |
| 8p23.1 microdeletion syndrome (ORPHA: #251071) | 1 (5.26%) | #76 |
| Chromosome 15q26-qter deletion syndrome (OMIM: #612626) | 1 (5.26%) | #74 |
| Prader-Willi syndrome (OMIM: #176270) | 1 (5.26%) | #109 |
| Angelman syndrome (OMIM: #105830) | 1 (5.26%) | #109 |
| Chromosome 15q13.3 deletion syndrome (OMIM: #612001 | 1 (5.26%) | #109 |
| Chromosome 18p deletion syndrome (OMIM: #146390) | 1 (5.26%) | #75 |
| Chromosome 3q29 microduplication syndrome (OMIM: #611936) | 1 (5.26%) | #99 |
| Chromosome 1p36 deletion syndrome (OMIM: #607872) | 1 (5.26%) | #107 |
| Chromosome Xp21 deletion syndrome (OMIM: #300679) | 1 (5.26%) | #171 |
| Chromosome Xp11.3 deletion syndrome (OMIM: #300578) | 1 (5.26%) | #171 |
| Chromosome Xq21 deletion syndrome (OMIM: #303110) | 1 (5.26%) | #172 |
| Chromosome 17p13.1 deletion syndrome (OMIM: #613776) | 1 (5.26%) | #263 |
